# Knowledge of Biobehavioral Organization Can Facilitate Better Science: A Review of the BioBehavioral Assessment Program at the California National Primate Research Center

**DOI:** 10.3390/ani11082445

**Published:** 2021-08-20

**Authors:** John P. Capitanio

**Affiliations:** California National Primate Research Center, University of California, Davis, CA 95616, USA; jpcapitanio@ucdavis.edu

**Keywords:** temperament, stress responsiveness, asthma, autism, anxiety, prenatal stress, ketamine, milk, behavioral inhibition

## Abstract

**Simple Summary:**

The BioBehavioral Assessment (BBA) Program was established in 2001 at the California National Primate Research Center to provide quantitative information on rhesus monkeys’ “intrinsic characteristics.” These characteristics such as temperament and stress responsiveness affect many aspects of an animal’s functioning and can be used to better manage the colony, and to select animals that are more homogeneous for research studies. Here, we review the BBA Program and describe how others have used this information in the design of their studies. We also describe results of studies aimed at understanding what experiences, both prenatal (e.g., exposure to stress) and postnatal (such as rearing), contribute to variation in intrinsic characteristics. The use of data such as these to identify subgroups of individuals with a greater risk for health-related outcomes is an animal model equivalent of a new trend in medicine, namely, precision medicine. Use of BBA data can also lead to a reduction in the number of animals needed in experimental studies.

**Abstract:**

Animals vary on intrinsic characteristics such as temperament and stress responsiveness, and this information can be useful to experimentalists for identifying more homogeneous subsets of animals that show consistency in risk for a particular research outcome. Such information can also be useful for balancing experimental groups, ensuring animals within an experiment have similar characteristics. In this review, we describe the BioBehavioral Assessment Program at the California National Primate Research Center, which, since its inception in 2001, has been providing quantitative information on intrinsic characteristics to scientists for subject selection and balancing, and to colony management staff for management purposes. We describe the program and review studies relating to asthma, autism, behavioral inhibition, etc., where the BBA Program was used to select animals. We also review our work, showing that factors such as rearing, ketamine exposure, and prenatal experience can affect biobehavioral organization in ways that some investigators might want to control for in their studies. Attention to intrinsic characteristics of subject populations is consistent with the growing interest in precision medicine and can lead to a reduction in animal numbers, savings in time and money for investigators, and reduced distress for the animals.

## 1. Introduction

Variation in nature is ubiquitous: trees vary in height, different bird species sing different songs, and tomatoes come in a variety of colors, shapes, and sizes. Darwin was perhaps the first to give careful thought to the idea of variation and the role it can play in evolution. It is significant that the first chapter in The Origin of Species [1] focused on plants and animals in a “captive” situation: The title of the chapter is “Variation under Domestication.” The very first paragraph sets the stage for the idea that decisions made by humans, through captive management/breeding programs, can influence basic and fundamental features of an animal’s behavior, biology, and structure, in ways that are beneficial for humans.

In the world of captive management of laboratory animals, the emphasis has generally been on keeping animals in uniform environments. From an experimentalist’s perspective, this is ideal: inferential statistics contrast variation between groups (one group received drug X and one group was administered a vehicle control) with variation within groups. One can find a statistically significant result by having greater variation between groups (i.e., a strong effect of drug X) and/or by reducing variation within groups, which can be achieved in a number of ways, such as by working only with a particular age/sex class of animals. Another way of reducing variation within groups is to provide uniformity in housing and husbandry conditions (although [2] argued that uniformity may adversely impact replicability). The assumption, under uniform housing conditions, is that the monkey in cage 7 is experiencing the same thing as the monkey in cage 31. However, is it? What if cage 7 is located in a top row within a housing room and cage 31 is on a bottom row? What if cage 7 is located near the doorway and cage 31 is located at the far end of the room? What if cage 31 is larger than cage 7? Some have suggested that factors such as cage location and size contribute to variation (e.g., [3,4], but see [5,6,7]). One might argue that these are simply more features of the environment that need to be controlled. However, what about the animal in cage 31 and the animal in cage 32? They may be of similar age and sex, and since they are housed next to each other, one can assume that they are getting very similar exposure to those (often uncontrolled) factors such as cage location and light intensity. However, the animal in cage 31 often seems “nervous” and may be experiencing repeated bouts of diarrhea, while the animal in cage 32 spends much of her time shaking the cage. What accounts for that variation? Can it affect experimental results?

It is our thesis that the difference between the two monkeys in cages 31 and 32 relates, very broadly speaking, to differences in the perceptions of the animals (that is, they perceive life in the housing room differently) and differences in coping abilities (passive vs. active coping, for example). Perception and coping are intrinsic characteristics of an animal—they are what the animal brings to each situation it encounters in the captive setting: a cage relocation and/or social separation for husbandry or scientific purposes, a change in the amount of available space (e.g., from an outdoor field cage to an indoor housing cage), encounters with unfamiliar animals (e.g., initial encounters during pairing, or when relocated indoors across from previously unfamiliar animals), husbandry routines, training for husbandry or scientific purposes, etc. Intrinsic characteristics are not immutable, but they are also not easy to change—among humans, for example, it might take cognitive-behavioral therapy to help the individual develop coping skills that might be more useful for their situation, and that the individual has not been able to change on their own. We have coined the phrase “biobehavioral organization” to refer to the more or less integrated functioning of animals’ psychological and physiological systems, and it includes intrinsic characteristics such as coping abilities, stress reactivity, temperament, hypothalamic–pituitary–adrenal integrity, cognition, and personality. Just as it is now common for managers of animal facilities to attend to, and standardize, captive housing conditions such as those mentioned above in order to have valuable and valid animal models, we believe it is equally important to consider aspects of individuals’ biobehavioral organization when assigning animals to research projects.

In 2001, we began our first year of collecting quantitative data on biobehavioral organization as part of our BioBehavioral Assessment (BBA) Program. The goals of the program were, first, to obtain data on aspects of biobehavioral organization; second, to make these data available to scientists for use in subject selection and for colony management personnel to use for better management; and, third, to explore the causes and consequences of this variation. In this review, we will describe the BBA Program and review how data from this program have been used in research. We will also review some of the work we have conducted identifying colony management practices that contribute to variation in biobehavioral organization, and which might also affect research results.

## 2. The BioBehavioral Assessment (BBA) Program

Details of the BBA Program have appeared in the many dozens of publications that have arisen from this program; an overview of the entire program and its many assessments can be found in [8]. Here, we describe the general features of the program and discuss the intrinsic characteristics that we assess in the program.

### 2.1. Subjects

The animals that we assess are infant rhesus monkeys (*Macaca mulatta*) that are between 90 and 120 days of age. We selected this age range because the animals are transitioning to eating solid food, have had limited social experience, enabling us to identify potential intrinsic characteristics of the animals that have had minimal shaping by experience, and are young enough to avoid layering our somewhat challenging assessment atop the normally challenging conditions the animals experience at a later age (e.g., ~6 months of age) as they become more completely weaned and their mothers resume cycling [9]. In fact, as our assessment involves a relatively short (25 h) relocation and separation from mother and/or peers, we conducted an extensive series of follow-up studies to demonstrate that there were no lasting consequences—behavioral, hormonal, immunological—of participating in the BBA Program [8]. We also wanted to keep the age window for assessments as narrow as possible, to minimize any age-related differences, although we have seen some age differences, even within this narrow window (e.g., [10])—these effects are typically small, however, and are evident largely because of the substantial sample sizes used in many of our analyses.

Animals are tested in cohorts of five–eight individuals, and they come from each of the four “colonies” at the CNPRC: (1) 0.2 hectare outdoor field corrals (FCR, field corral-reared) that each house up to 200 animals of all age/sex classes; (2) outdoor corncrib (CCR, corncrib-reared) structures that each house up to 30 animals [11]; (3) the indoor colony, comprising females on time-mate protocols, in which infants are housed in standard-sized cages with their mothers and, in the majority of cases, one additional adult and infant pair (IMR, indoor mother-reared), which facilitates monitoring of reproductive cycles; (4) our indoor nursery (see also Section 3.2.1), in which animals are relocated on the day of birth and are individually housed in incubators until 3 weeks of age, at which point they are given visual access to an infant of the same age with whom they are subsequently paired at 5 weeks of age (NR, nursery-reared). Months after BBA testing, IMR and NR animals are housed in corncribs with other monkeys that were similarly reared and may eventually form new groups in a field corral. As it might be expected, and as we will describe below, rearing history is a significant contributor to variation in biobehavioral organization.

### 2.2. Procedures

Animal care staff deliver the infants to our testing area by 0900 h. FCR and CCR mothers are also captured with the infants and are housed in an area of our facility that is out of sensory range of the infants. The infants are weighed upon delivery and are placed in individual, standard-sized female cages that each contain a towel, a stuffed toy, and a novel object (see later section). Other animals in the testing cohort can be heard and smelled but cannot be seen during the 25 h period. Food and water are available ad libitum. After fifteen minutes of habituation, assessments begin and are always conducted in the same sequence for all animals. Animals within a cohort are tested in a pre-determined random order that remains constant for all assessments. Testing ends around 1630 h on day 1 and re-commences at 0700 h the next day. Infants are returned to their mothers (FCR, CCR, IMR) or pair-mate (NR) at 1000 h; infants from the field cages and corncribs remain with their mothers for an hour in the mothers’ holding area to allow for nursing, after which the mother and infant are returned to their original cages.

### 2.3. Assessments

Here, we describe our assessments in terms of what they are aiming to tell us about the animals and briefly describe how the data are obtained. For more details, see [8]. A timeline for the assessments is shown in Table 1.

#### 2.3.1. Responsiveness and Adaptation 

For the overwhelming majority of animals, particularly the FCR and CCR animals, enrollment in the BBA Program involves their experiencing, for the first time, (a) a separation from mother and other companions and (b) a relocation into an indoor housing area, which involves, among other things, a change in illumination and the light/dark cycle, and a reduction in available space. How do the animals respond to these changes, and is there variation in this responsiveness? Do animals adapt to the situation over time? These are important questions that are relevant across the animals’ lives, as they will continue to experience relocations, indoor housing, and separations from companions. To look into these characteristics, we conduct five-minute focal animal observations on the animals 15 min after their arrival (0915 h on day 1), and then again at 0700 h on day 2. Exploratory and confirmatory factor analyses (described in [12]) of the recorded behaviors identified two latent traits, labeled Activity and Emotionality, underlying the behavioral responses. The day 1 Activity and Emotionality measures tell us something about how responsive the animals are to the separation and relocation, and the day 2 measures tell us about adaptation. In fact, we do see wide variation in both domains: while a predominant response on day 1 is for watchful wariness and low activity, we also see some animals vocalizing repeatedly and engaging in considerable activity. Similarly, on day 2, most animals that were relatively inactive on day 1 become more active, suggesting good adaptation, while others remain relatively immobile and wary. These Activity and Emotionality measures also define for us a pattern of behavior labeled behavioral inhibition (also known as inhibited temperament)—animals that are below the mean for Activity and Emotionality on day 1, as well as below the mean for Activity and Emotionality on day 2, are defined as behaviorally inhibited [13,14].

#### 2.3.2. HPA Regulation

One of the body’s major stress response systems involves the hypothalamic–pituitary–adrenal (HPA) axis, and its principal output is cortisol, a glucocorticoid hormone that can be measured easily in peripheral blood. The HPA axis is a regulated system: under normal conditions, corticotropin-releasing hormone (CRH) is released from the hypothalamus and travels to the anterior pituitary where adrenocorticotrophic hormone (ACTH) is released. ACTH then travels via the circulation to the adrenal cortex, where cortisol is released. Cortisol travels throughout the body, including to the hypothalamus and pituitary, where it can dampen further release of CRH and ACTH through the process of negative feedback. Sometimes, this system is dysregulated; negative feedback does not seem to work, or the normal diurnal secretory pattern of cortisol (high levels around awakening time that decline over the course of the day) is altered. Changes such as these characterize many human cases of depression and post-traumatic stress disorder [15]. We assess cortisol concentrations at four time points during the 25 h BBA: at 1100 h and 1600 h on day 1, and at 0830 h and 0900 h on day 2. The first sample reflects the HPA response to the relocation and separation, and the second sample suggests whether the animal has adapted to the situation. Immediately after the second sample is drawn, animals are given a standardized dose of dexamethasone, a synthetic glucocorticoid with a high affinity for glucocorticoid receptors. The dexamethasone suppression test is a clinical test designed to assess the integrity of the HPA system, particularly negative feedback. Our third blood sample, at 0830 on day 2, should show suppressed cortisol output as a result of the dexamethasone administration. Following this sample, animals are injected with a standard dose of ACTH to examine adrenal responsiveness in a fourth sample that is taken 30 min after ACTH administration. While glucocorticoids’ principal function is influencing glucose concentrations in the blood, an important secondary function involves regulation of immune responses, particularly inflammation. A dysregulated HPA axis, then, may have implications for physical health as well as mental health.

#### 2.3.3. Social Challenges

Animals in a captive primate colony may experience social challenges at multiple points in their lives: new social groups may be formed that contain previously unfamiliar animals; indoor animals may be paired with novel animals; or even if animals are housed individually, a relocation to a new room, or a new cage in the same room, may involve establishing relationships with animals housed across the aisle. Moreover, humans may be perceived as a challenge, such as when a veterinary tech is visually evaluating an animal in its cage—the staring of the human may be interpreted by the monkey as a threat. We record behavior on our animals in two situations that tap into social challenges. The first is a video playback procedure, where we show a video of an unfamiliar adult male monkey alternately displaying bouts of aggressive and neutral behavior. Through the use of video, we are able to present to the animals a reasonably realistic, and standardized, conspecific stimulus, and we can assess how animals’ responses differ depending on whether they are viewing aggressive segments or neutral segments. Our second challenge is a human intruder challenge. In this case, the technician presents her face in profile for one minute from ~1 m from the monkey, after which she moves closer (~1/3–1/2 m), in the same orientation, for a second minute. Then, returning to the far position, she stares at the animal for a minute before moving to the near position for the fourth minute. Others have developed similar human intruder tests, though with some different details [16]; since our version incorporates a graded series of challenges from least distressing (profile orientation from a far position) to most distressing (stare orientation from near position), we can assess the extent to which animals differentiate the varying conditions of threat.

#### 2.3.4. Temperament

As described above, behavioral inhibition is one dimension of temperament that we assess, and it is defined behaviorally. Other measures are obtained at the end of the 25 h biobehavioral assessment, when the technician rates each animal using a 16-item instrument designed to assess other aspects of temperament. Exploratory and confirmatory factor analyses, reported in [12], revealed four dimensions: Confident (what others call “bold”), Gentle (which seems to reflect a passive coping style), Nervous (similar to the human personality dimension of neuroticism), and Vigilant (a temperament factor that, to our knowledge, has not been reported before in any species but is not unexpected in a species with a strong hierarchical structure, in which animals must play close attention to the ranks of animals in their vicinity). These ratings are assigned out of sight of the animals in order to prevent the current behavior of the animal from having a disproportionate influence on the ratings, and with a goal of obtaining overall “thumbnail” assessments of the animals’ behavioral characteristics during the entire period of testing. This includes not only how the animals behaved during the various assessments, but also how they responded during feeding, hand catching (both for phlebotomy and temporary relocation to a test cage for some of the other assessments), blood sampling, and when other animals were being handled for the same procedures.

#### 2.3.5. Response to Novelty

Animals’ willingness to engage with novelty is often used to index aspects of temperament (e.g., impulsivity [17]). While most aspects of BBA testing are novel to all of the animals, we included a novel object in the animals’ holding cages to assess their willingness to engage with an object that is relatively small (a cylinder measuring 9 cm long × 3.8 cm in diameter) and that can be held in their hands and placed in their mouths. Inside each novel object is an actimeter that records any force exerted on the object. At the end of the first day, the original novel object is swapped out with another to maintain the novelty. At the end of the 25 h period, the data from both objects are downloaded and summarized to indicate how frequently the animals moved the objects.

#### 2.3.6. Memory

We wanted to include a measure of cognitive function, but most such tasks involve training which, in the context of our 25 h assessment, was not feasible. Instead, we settled on a visual paired comparison task which involves no training and is a test of recognition memory. This test is sensitive to a variety of adverse experiences, including damage to limbic structures [18], prenatal exposure to methyl mercury [19], and high-risk pregnancies and births [20]. Each monkey sees a pre-recorded video that presents seven problems. For each problem, the subject is presented with identical pictures side by side on a monitor for 20 s, after which the screen goes blank. Next are two 8 s test trials, in which the now-familiar picture and a novel picture are presented; the two trials differ only in the placement of the stimuli. The typical response of young monkeys (and humans) is to spend more time looking at the novel picture in the test trials, and the outcome measure is the proportion of total looking time that animals spend looking at the novel stimuli (i.e., duration of looking at novel divided by duration of looking at novel + familiar stimuli). Our implementation of this test has two important differences from others’ use. First, our animals are unrestrained—animals can choose to look or not, which was why our outcome measure is based on the amount of time each animal did in fact look at either stimulus during the test trials. Second, our stimuli are explicitly not neutral objects but, rather, are photos of unfamiliar conspecifics with neutral expressions. The goal was to use stimuli that had some ecological relevance to the animals.

#### 2.3.7. Potential Moderators of Experience

We also record information that can be used to look statistically for interactions—that is, subgroups of animals may be affected differentially based on some of these characteristics. One thing that is recorded on all animals is rearing history, as described above: field corral, corncrib, indoor mother, or nursery. Sex and age at time of BBA testing are also recorded, as well as the weight of the animals at the time of testing. Indoor-reared animals, particularly NR animals, typically weigh more than FCR animals, and weight can influence, for example, cortisol concentrations. Specific pathogen-free (SPF) status is also recorded. Animals are considered SPF if they are free of four endemic viruses [21]. Our SPF colony was created by removing infant monkeys on the day of birth and rearing them in a nursery, as described above. Eventually, SPF animals are formed into corncribs or field corrals. Testing continues to ensure the animals remain SPF, but as they reproduce, their offspring are themselves SPF, meaning that nursery rearing is no longer needed. For FCR infants, we also record the ranks of their mothers. A low social rank, for example, could be a stressor [22], which could affect fetal development. We also record whether the infants were fostered. Any breeding facility with multiple cages has to have a plan to move genes between cages. At our facility, an infant from one cage is swapped with an infant in a different cage. This is usually carried out on the day of birth, with a goal of reducing inbreeding in cages.

Finally, we genotype the animals for two genes of neuropsychiatric interest. One gene codes for the serotonin transporter (5-HTT), the molecule responsible for reuptake of serotonin from the synaptic cleft following its release. A polymorphism in a promoter region of this gene (*5-HTTLPR*) affects the transcription of the transporter protein, with a short allele showing reduced transcriptional efficiency compared to the long allele [23]. Animals with at least one short allele showed greater emotionality compared to long/long homozygotes [24]. The second gene of interest is the X-linked monoamine oxidase-A (*MAOA*) gene, the protein product of which deaminates the monoamine neurotransmitters. This gene also has a polymorphism in its promoter region (*MAOA-LPR*), consisting of a variable number of repeat sequences, and this polymorphism has been related to variation in transcriptional efficiency of the gene in both humans [25] and rhesus monkeys [26]. The low-activity allele has been associated with poor affect regulation [27]. For both genes, the alleles that promote reduced transcriptional efficiency were initially considered “risk” alleles, insofar as individuals that experience some form of adversity showed poorer behavioral outcomes if they also had the low-transcriptional variants of the genes (e.g., [28]). Growing evidence, however, suggests these genes may be viewed more productively as “sensitivity” or “plasticity” alleles: under adverse conditions, they typically confer a greater risk for poor outcomes, but data also suggest that under extremely beneficial conditions, they promote more positive outcomes [29,30].

## 3. How Can Knowledge of Animals’ Biobehavioral Organization Facilitate Better Science?

The idea that individual characteristics of animals can be important contributors to scientific outcomes is not new; as described above, a statistically significant result for an experimental study is more likely if individual variation within groups can be minimized, and one way to ensure that might be to select animals with similar characteristics. Historically, though, the phrase “similar characteristics” has typically referred to gross physical or demographic qualities: age, sex, living in the same room, etc. Within the last decade or two, however, the phrase “similar characteristics” has been broadened considerably as part of a new focus in research on health and disease on personalized (or precision) medicine. This approach was promoted in 2006 by Dr. E. Zerhouni, then-Director of NIH, in his testimony to Congress on his “3Ps” approach to health: “Because we now know that individuals respond differently to environmental changes according to their genetic endowment and their own behavioral responses, we can envision the ability to precisely target treatment on a personalized basis [31] (p. 2, emphasis added).” Five years later, the National Research Council described precision medicine as including “the ability to classify individuals into subpopulations that differ in their susceptibility to a particular disease, in the biology and/or prognosis of those diseases they may develop, or in their response to a specific treatment. Preventive or therapeutic interventions can then be concentrated on those who will benefit, sparing expense and side effects for those who will not [32] (p. 125, emphasis added).” Identifying subpopulations has been one major use of BBA data. As such, the BBA Program contributes strongly to the goal of the second of the 3Rs, namely, a reduction in animal numbers [33] (see below).

It is worth noting a caveat at this point. In inferential statistics, one studies a sample of individuals to find answers that one hopes will generalize to the broader population. Inferential statistics assume that subjects were sampled randomly, which would then lead to broad generalization of the results from that sample. If work is conducted with subsamples, that is, individuals who are selected based on certain characteristics, is this not a violation of a basic assumption of inferential statistics? The answer is yes, but only with respect to generalizability—the results obtained from samples that shared some risk factor that identifies a subgroup (e.g., a genetic predisposition toward cancer) would generalize to others with that risk factor, but not to individuals that do not possess the risk factor. Therefore, the concern with not having a random sample is not about the statistical procedures per se, but rather about who the results of the analysis would generalize to. However, we would argue this is always an issue. Individuals that work with nonhuman primate models often do not obtain a random sample of their animals. “Random sampling” implies that all animals within the broad group of interest (e.g., adult male monkeys) have an equal likelihood of being selected for a study. This is most certainly not the case at many, if not all, primate facilities. For example, at our facility, someone wanting to study the effects of a drug on a pathogen will never receive alpha males from our outdoor field corrals assigned to their projects—relocating such animals for assignment to a project could cause disruption to the groups, and potential injury to other animals. Not being able to obtain alpha males may not be a problem for the particular process under study (i.e., rank may have no impact on the efficacy of drug X anyway), but it should definitely limit the generalization of the results. It is our position that data from the BBA program can not only provide useful information for selecting subgroups for study but can also provide a set of measures of intrinsic characteristics that can be used to identify the population to which one’s results might generalize, even if one is not explicitly studying subpopulations.

### 3.1. The BBA Program Has Been Used to Define Subsets of Animals for Investigation

#### 3.1.1. Asthma

**Figure 1:**
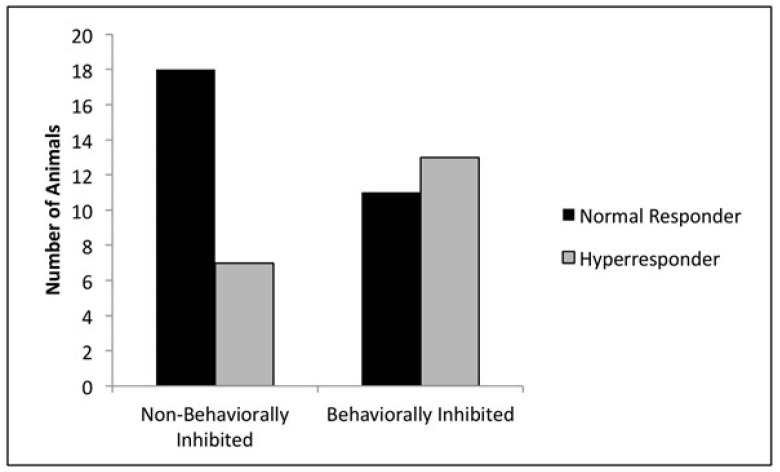
Inhibited temperament style and the airway response. Inhibited animals are significantly more likely to show an exaggerated airway response (i.e., to be a hyperresponder) compared to non-inhibited animals. Reprinted from [37].

Asthma has long been recognized as a disorder that has a significant psychosocial component: an early study [34] described children with asthma as having a temperament pattern described as “slow-to-warm-up”—children that are shy or inhibited in novel situations. Other studies, both retrospective and prospective, have also shown that anxiety, depression, and/or neuroticism (which are themselves linked to an earlier inhibited temperament style) confer risk for asthma. The California National Primate Research Center (CNPRC) is the only NPRC with a Respiratory Diseases unit, and an active set of researchers are engaged in understanding, and designing new treatments for, asthma. A principal outcome measure in these studies is airway hyperresponsiveness (AHR), which is a significant component of asthma—through careful administration of varying doses of an adrenergic agonist, such as methacholine, which constricts the bronchial airways, followed by measurement of airway resistance, one can determine the amount of methacholine that leads to a standard increase in resistance. Individuals that need very little methacholine to produce a 200% increase in airway resistance can be said to show AHR. Investigators using this monkey model to study the pathology and test new treatments for asthma really want to study animals that display AHR, but at our facility, only 20–25% of the animals show AHR [35]. We first conducted a retrospective study contrasting animals that showed AHR with those that did not [36] and found a pattern of inhibited biobehavioral style that differentiated the groups; animals that showed AHR showed low Emotionality on day 1 of our responsiveness observations; high scores on Vigilant temperament; and a blunted cortisol response for our first two samples. We next used this information to prospectively select individuals with this profile and test their airways [37]. The prospective study confirmed our retrospective results and showed that, whereas simply selecting animals in our field corrals will lead to 20–25% of the animals showing AHR, using our BBA-derived criteria can increase this number to approximately 50% (Figure 1). This results in considerable savings of time, money, and animal distress by only having to screen twice the number of animals needed for a study, instead of four times the desired number. Since the publication of these results, three different research groups have utilized our measures to select subjects at risk for AHR to understand the mechanisms involved in the response, as well as for trying new treatments (e.g., [38]).

#### 3.1.2. Autism Spectrum Disorder (ASD)

**Figure 2:**
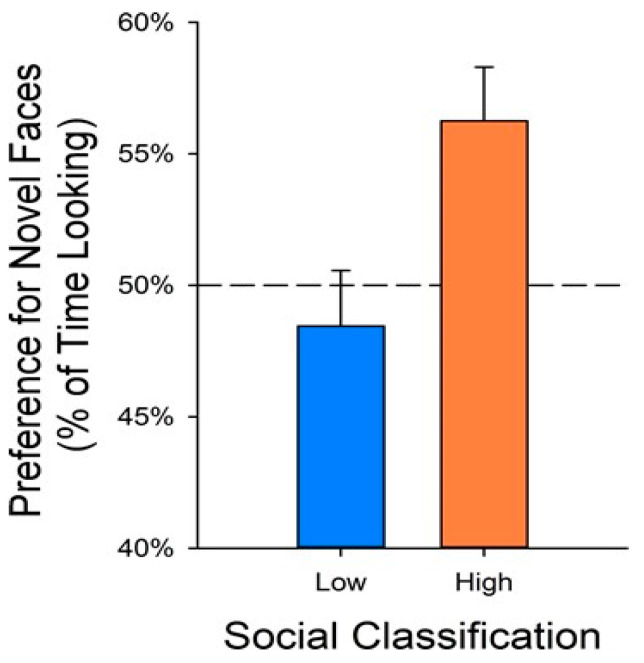
Novelty preference and social classification. Animals identified as highly social were more likely to detect novel from familiar faces in the memory task. Reprinted from [40].

A core feature of ASD is a deficit in social interaction and social communication, including difficulties in developing and maintaining social relationships and in adjusting one’s behavior to various social contexts [39]. To date, however, there are no approved medications to treat these deficits. Working with K. Parker from Stanford University, we observed the social behavior of 168 juvenile males (1–4 years old) and classified the top 25 and the bottom 25 based on the amount of nonsocial behavior they displayed, in an effort to determine if naturally occurring low sociality could be a model for the social deficit in ASD. We typically observe animals in cohorts of 6–10 animals, for a two-week period for each cohort. Thus, observations of 168 animals represent a significant investment of observer time. Since all animals had participated in the BBA Program at 3–4 months of age, we were curious whether there were potential BBA predictors of social functioning. If so, the BBA Program could serve as a high-throughput screening tool to identify animals at an early age before the social deficits appear, and to treat the animals to possibly prevent the deficits from occurring at all. Our focus was on the two conspecific “social” tests: the video playback social challenge, and, because we used pictures of unfamiliar monkeys as stimuli, the test of visual recognition memory. We found significant differences between the low social (LS: those animals with high values for nonsocial behavior as juveniles) animals and high social (HS: animals with low values for nonsocial behavior) animals for measures from these two tests. In fact, three measures perfectly predicted the social classification for these 50 animals: the ratio of looking at the aggressive segments relative to the neutral segments during the video playback; the ratio of gaze aversion to the aggressive relative to neutral segments in the video playback; and the preference for the novel stimuli in our memory test (Figure 2). For all three measures, the LS animals had significantly lower scores than the HS animals [40]. In other work, we found that LS animals had significantly lower concentrations of arginine vasopressin (AVP) in their cerebrospinal fluid compared to HS animals [41]. These data suggested a treatment trial in which BBA data were used to identify animals that might be at risk for poor social outcomes. Animals were selected based on their BBA data and were administered intra-nasal vasopressin in two doses (along with a vehicle control). They were then tested on the same task for visual recognition memory to determine if their responses improved. Data are still being analyzed, but preliminary evidence suggests the treatment may have been successful.

#### 3.1.3. Behavioral Inhibition (BI)

**Figure 3:**
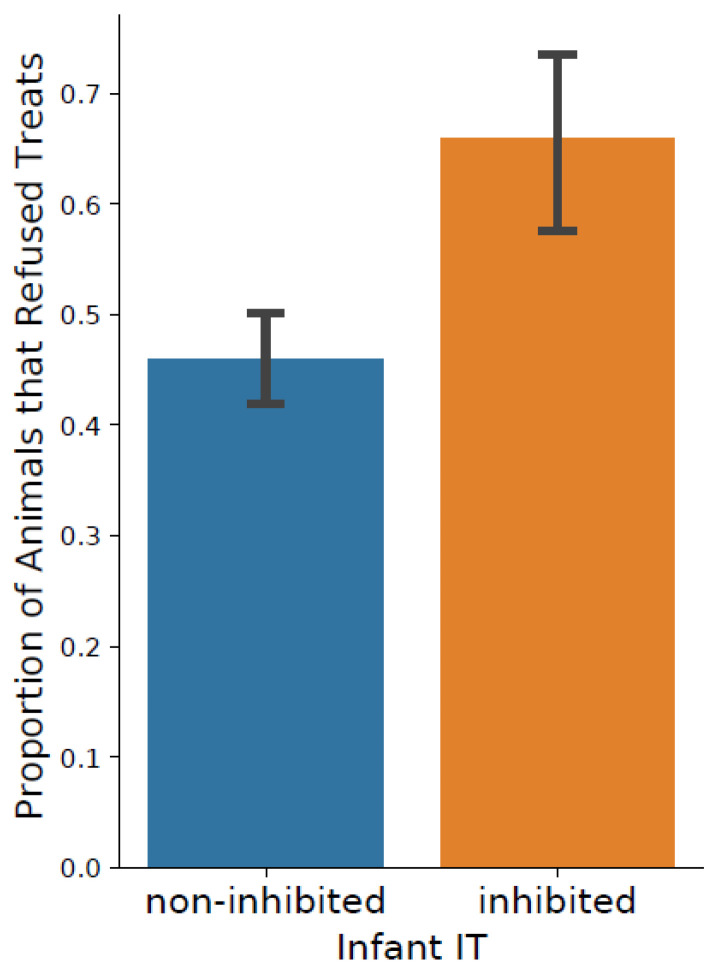
Behavioral inhibition and food retrieval. Behaviorally inhibited animals, identified years earlier during the BBA Program, are significantly more likely to refuse a treat compared to non-inhibited animals. Reprinted from [14].

Children that are behaviorally inhibited are timid in situations involving novel people or objects. BI, also known as inhibited or anxious temperament, in childhood is a major risk factor for later anxiety and depression [42], and its prevalence in children has been estimated at 15–20% [43]. (Elsewhere [13], we note that this is a different concept than the inhibited biobehavioral style referred to above for the studies of asthma.) Interestingly, the definition of BI used in the BBA Program (below the mean for day 1 Emotionality and Activity, and day 2 Emotionality and Activity) reveals a prevalence for BI of 18.3% among more than 5000 infants tested in the BBA Program, comparable to the prevalence seen in the human population. Two research groups have utilized BBA data to examine the genetic, behavioral, and neuroanatomical correlates of BI. D. Fox et al. [14] reported that animals identified as behaviorally inhibited at 3–4 months of age were significantly more likely to refuse a preferred treat given by an unfamiliar person up to several years after their BBA assessment (Figure 3). Fox et al. [14] also reported that BI is heritable (h^2^ = 0.19), and a genome-wide association study identified a gene, *CTNNA2*, that has been identified in human association studies of anxiety disorders. The gene encodes neuron-specific catenin expressed throughout cortical and non-cortical structures and suggests a molecular mechanism for further study. A second research group, headed by P. Lavenex, examined the structural differences in the brain in a small group of BI and non-BI animals [44]. Inhibited monkeys had altered volumes in areas of the hippocampus and amygdala, results that were consistent with those found in other animal models and in imaging studies with humans. These studies strongly suggest that this model of BI has many significant overlapping features with what is known of this phenotype in humans. The routine identification of BI as part of the BBA Program means investigators do not have to assess five animals to identify one inhibited animal, again saving time, money, and distress for the animals.

#### 3.1.4. Temperament and Mother’s Milk

**Figure 4:**
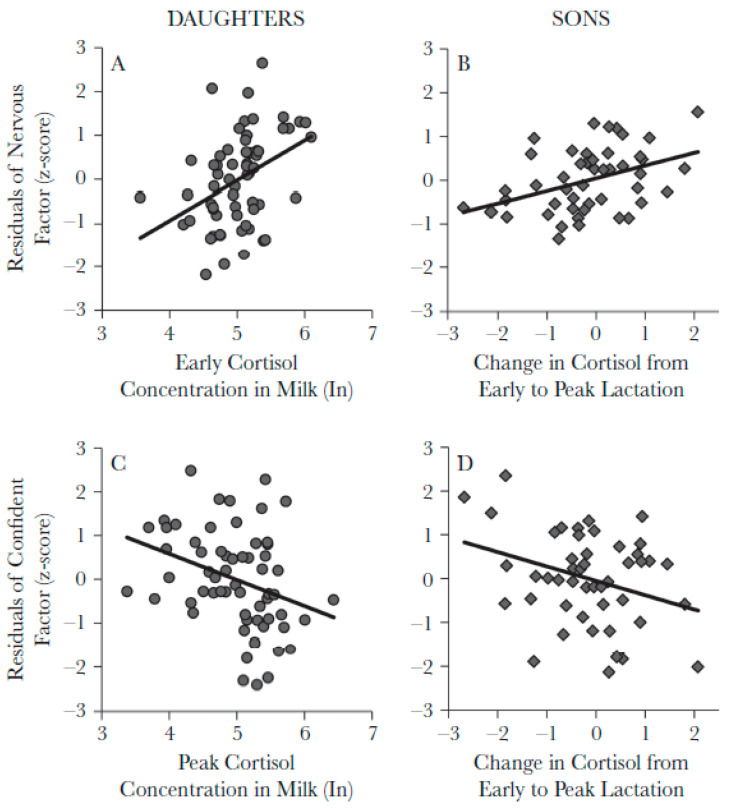
Milk cortisol concentrations and temperament. For daughters, cortisol concentrations during early (**A**) and peak (**C**) lactation are associated with Nervous and Confident temperaments, respectively. For sons, the change in milk cortisol concentrations from early to peak lactation was associated with Nervous (**B**) and Confident (**D**) temperaments. Reprinted from [45].

Parental care varies widely in the animal kingdom, but one defining characteristic of mammals involves specific investment by mothers in providing nourishment to their offspring. There is growing realization, however, that mammalian milk contains more than just nutrients; rather, it contains substances that serve as a form of communication between mother and offspring, providing signals to the infant about the mother’s, and hence the infant’s, broader world. One component of milk that is particularly important is the concentration of glucocorticoids (GCs) present. A study by K. Hinde and colleagues [45] utilized BBA-assessed animals to examine the roles of milk GC and milk energy density in temperament and growth in n = 108 infants. Glucocorticoid concentrations in mother’s milk were associated with temperament—daughters whose mothers had higher concentrations early in lactation (at 1 month of age) were more Nervous, and daughters whose mothers had higher concentrations at peak lactation (at ~3.5 months of age) were less Confident. For sons, greater changes in cortisol concentrations from early to peak lactation were also associated with more Nervous and less Confident temperaments (Figure 4). These results were independent of any effects of available milk energy. Lower parity mothers were among those with high GC concentrations in milk, and higher concentrations were also related to greater infant weight gain. These investigators proposed that high maternal GCs in milk “program” infants to have a more cautious phenotype (i.e., a high Nervous temperament), and to prioritize growth at the expense of exploration, play, and boldness (i.e., a low Confident temperament). Having established a relationship between maternal milk GC concentrations and temperament, the next step was to understand how milk GCs affected brain development. Using BBA data, these investigators identified “High Nervous, Low Confidence” and “Low Nervous, High Confidence” animals for participating in a neuroimaging study to better understand the neural regions involved in this temperament effect. Data from this study are currently being analyzed.

#### 3.1.5. Trait Anxiety and Reproduction

Investigators from Cincinnati Children’s Hospital were interested in studying the joint role of trait anxiety, inflammation, and prenatal stress on preterm birth. The relevant issue for the present discussion is that the investigators were interested in *trait* anxiety specifically, not anxious behavior that is seen as a result of an acute experience (which is state anxiety). As trait anxiety is a style of behavior that is intrinsic to the animal, we identified trait-anxious adult females if they (a) had a low affective output in our responsiveness observations during BBA, and (b) evidence of anxious responding in food retrieval [14] and human intruder tests conducted when the animals were adults. Our expectation was that animals that were anxious as infants and were still anxious as adults were probably animals with trait anxiety. Having the BBA data on animals when they were younger allowed us to adopt this strategy, rather than a strategy where the distinction between state and trait anxiety might be less clear, if we only had test data on animals when they were adults.

### 3.2. What Factors Contribute to Variation in Biobehavioral Organization?

During the two decades that the BioBehavioral Assessment Program has been in existence, we have conducted a number of analyses focused on how variation in biobehavioral organization arises. This information may be of interest to colony management personnel, but it is also valuable for the experimentalist who is interested in selecting animals or in balancing groups within an experiment. Here, we briefly describe these studies.

#### 3.2.1. Rearing

In trying to understand what contributes to variation in biobehavioral organization, rearing is the “low-hanging fruit.” We have known for decades how isolation/nursery/restricted rearing can impact behavior [46,47] and physiology [48,49,50]. This information has been valuable for designing new approaches to rearing protocols that mitigate at least some of the adverse effects. For example, for several years at our facility, monkeys were reared in a nursery from the day of birth in order to derive our SPF colony (described above). These days, animals are nursery reared (NR) only for experimental purposes, or if an infant is abandoned and cannot be fostered. One obvious change in the NR protocol was to provide a pair-mate, as soon as it was deemed safe, to facilitate social and emotional development. These efforts have been successful, in that NR animals at our facility are generally psychologically healthy. However, there remain enough biobehavioral differences due to rearing that investigators should certainly consider rearing history when selecting animals.

We reported rearing differences in several papers. Not surprisingly, some of the biggest differences were between NR animals and those from the other three rearing conditions: field cage reared (FCR), corncrib reared (CCR), and indoor mother reared (IMR). NR animals were less active in several of the BBA assessments, compared to animals in the other three rearing conditions [51]. Moreover, NR animals showed less differentiation between conditions for some of the assessments; for example, during the four conditions of the human intruder (HI) task, NR animals had consistently low levels of activity, while FCR and CCR animals’ responses varied more based on condition. Interestingly, IMR animals also showed less differentiation between conditions during the HI test, but unlike the low levels seen in the NR animals, the IMR animals had high and consistent levels of activity. Three further sets of results from our analysis of rearing effects deserve note.

The first set of results examined aggressive, fearful, and anxious behaviors during our human intruder social challenge [52]. We were especially interested in whether the genotype for the monoamine oxidase-A promoter (*MAOA-LPR*) could moderate the effect of rearing. Several genotype-by-rearing interactions were found; for example, CCR animals were more likely to display threat behavior overall in the high-challenge conditions (the stare-near and stare-far conditions), compared to the other groups, but animals with the sensitivity allele for *MAOA-LPR* displayed the highest level. In contrast, IMR animals with the sensitivity genotype showed significantly more yawn, an anxious behavior. Interestingly, despite having about 10 times the number of animals in the FCR sample (n = 375) compared to the other three rearing groups, the FCR animals showed no genotype effects on behavior in the HI test. This suggested to us that rearing in rich, species-typical social groups may buffer individuals from a genotype that, given other circumstances, might be a risk factor for poor behavioral outcomes. The important point of these results is that simply examining the rearing histories of one’s prospective subjects may not be enough to ensure homogeneity. To be sure, IMR and NR are unusual rearing conditions, but some animals are more affected by these conditions (i.e., animals with the low-activity, sensitivity, *MAOA-LPR* genotype) than others.

The second set of results involves significant and substantial rearing differences in HPA regulation among NR animals [10]. Recall from above that we assess cortisol concentrations on four occasions during the 25 h BBA. Among FCR and CCR animals, cortisol tends to increase from samples 1 to 2; dexamethasone administration leads to lower concentrations for sample 3, and ACTH injection leads rapidly to elevated levels within 30 min. NR animals showed no change, or sometimes a decline in cortisol, from samples 1 to 2. More significant, however, was that cortisol concentrations for NR animals for all four samples were significantly lower than for animals from the other rearing conditions—it is as though the regulated HPA system in NR animals has a lower set point than the case for FCR, CCR, or IMR animals. This result suggests that the regulation of the HPA axis in NR animals is different. Given the role that glucocorticoids can have on other biological functions, such as immunity, inclusion of NR animals in a study could lead to greater variability in some disease-related outcome measure, for example.

The third set of results indicates that the effects of rearing are not confined to the generation that experienced unusual rearing. E. Kinnally made use of our center’s strategy of nursery rearing for generating SPF animals to see whether the offspring of these NR dams and sires showed evidence of their parents’ NR experience. In our first study [53], offspring were themselves NR and therefore had virtually no postnatal experience with their dams or sires. Those animals that had sires (but not dams) that were NR showed significantly more Emotionality (from the responsiveness and adaptation observations) and higher cortisol concentrations. We followed this study up with one in which the animals were born and reared in the field corrals and examined whether, under those conditions, there was still an influence of dam or sire rearing history [54]. We also examined the rearing histories of the animals’ grandparents and great-grandparents. Field cage-born animals whose fathers were NR had significantly higher values for Nervous temperament, and significantly lower immune cell numbers (which were assessed from the first blood sample taken during BBA). This was also true for paternal grandfather rearing—animals born in the field cages whose paternal grandfathers were NR had a more Nervous temperament, lower immune cell counts, and lower cortisol. Finally, effects of paternal great-grandfather NR were evident for Nervous temperament. Together, these data indicate that any evaluation of “rearing” for subject selection should consider how sires and grandsires were also reared.

#### 3.2.2. Ketamine Exposure

Ketamine is an N-methyl-D-aspartate (NMDA) receptor antagonist that is commonly used in primate laboratories for immobilization and anesthesia. While ketamine’s primary action is on glutamate systems in the central nervous system, it has long been known that ketamine administration affects monoamine neurotransmission as well: research in rats, for example, demonstrated that after a single ketamine injection, brain levels of epinephrine, serotonin, dopamine, and norepinephrine were significantly altered [55,56]. This led us to wonder whether doses administered in the clinical range (e.g., 10 mg/kg) might have an impact on behavior that is moderated by *MAOA-LPR*, a gene that regulates monoaminergic neurotransmission. We identified n = 82 infants that had known dates of conception, as they were part of our colony’s time-mate program (these animals would become IMR animals once born) [57]. Each animal had one–nine exposures to ketamine in utero, for routine health checks, minor procedures (e.g., dental cleaning in dams), or ultrasound exams to track fetal development. Exposures were classified as occurring in the first, second, or third trimester of pregnancy. The results indicated that animals with the sensitivity *MAOA-LPR* genotypes were most affected by ketamine, while animals with the other genotypes were mostly not affected. Effects were especially evident for animals exposed in the first and second vs. third trimesters. Animals with greater exposure in the first trimester (and who had the sensitivity genotype) showed a pattern of inhibited behavior—lower Emotionality in the responsiveness observations and reduced contact with novel objects—while animals with the sensitivity genotype and who were exposed in the second trimester showed a positive relationship between the number of exposures and Activity, as assessed in the responsiveness observations.

Our study involved animals that experienced unusually high levels of ketamine exposure—not surprising since animals were bred for participation in fetal experiments, and development needed to be tracked (via immobilization of the mother) frequently before assignment to a project. We wondered whether lesser exposures might have an impact, and whether rearing in a more naturalistic environment might mitigate some of the adverse effects. Consequently, we examined health records of n = 408 FCR animals and classified them by whether they had one ketamine exposure prenatally and/or one ketamine exposure postnatally (but before we tested the animals in the BBA program, at 3–4 months of age) [58]. Prenatal exposures were further classified by trimester, based on estimated conception dates (birth date minus 165 days). We confirmed that animals exposed in the second trimester showed higher Activity in the responsiveness observations, although this was a main effect, and not an interaction with *MAOA-LPR*. In our test of visual recognition memory, we found that animals with the best scores were those with no prenatal exposure, with one exception: animals that had one exposure postnatally before 3 months of age had worse performance on our test of visual recognition memory, responding at chance levels. Other evidence suggested impaired performance on this task for most animals that had a prenatal exposure.

It is important to note that our effect sizes were relatively small for our studies and were nothing like the effects seen in studies where animals are given continuous doses for hours in order to mimic use of ketamine in pediatric populations (e.g., [59]). Nevertheless, our data indicate that ketamine exposure is a contributor to variability in affective behavior, activity, and cognition. Studies examining these endpoints might benefit from examination of the ketamine exposure history—prenatal and postnatal—of their subjects.

#### 3.2.3. Prenatal Effects

The prenatal period is a time of major development of organ systems, including the brain which is largely responsible for behavior. In addition to examining the role of ketamine during the prenatal period, we have also found three other factors which, when experienced prenatally, contribute to variation in biobehavioral organization. We review each briefly here.

Maternal obesity. There is growing evidence that maternal pre-pregnancy body mass index and gestational weight gain are associated with neurodevelopmental disorders in offspring (see numerous references in [60]). We identified n = 173 IMR infants that met inclusion criteria and assessed their BBA outcome measures against their dams’ pre-conception body condition score, and dams’ gestational weight gain. Offspring of mothers with a greater baseline body condition score and/or gestational weight gain exhibited a pattern of poor adaptability characterized by greater Emotionality in the responsiveness observations, blunted affective response to a human intruder social challenge, lower cortisol levels following dexamethasone administration, lower Confident temperament, and reduced performance in our memory task.

Wildfire exposure. Animals comprising our breeding colony (i.e., FCR and CCR rearing) live outdoors in large enclosures year-round and therefore are exposed to all of the ambient weather and climate factors common to the Sacramento Valley in California. One of these factors is air quality, which is affected by the growing number of wildfires in the state. In November 2018, California’s deadliest wildfire discharged a plume of smoke that blanketed the valley for two weeks. It was unusual to have a fire of this magnitude that late in the season, but this one occurred in the middle of the breeding season. We were interested in whether exposure prenatally might have an impact. We [61] studied n = 89 FCR animals, n = 52 of which had estimated conception dates before or during the smoky period. The remainder (n = 37) were conceived after the smoke had cleared. Exposed, compared to non-exposed animals, had higher levels of inflammation (C-reactive protein is routinely assessed from the first blood sample in BBA), blunted cortisol responsiveness, more passive behavior, and deficits on our visual recognition memory task. Parallel analyses on nearly 2500 animals in a historical control cohort showed that none of these effects were due simply to the timing of conception.

Prenatal stress. While life in our facility’s 24 0.2-hectare field corrals is generally peaceful (for rhesus monkeys), there are occasions when aggression escalates and results in a matrilineal overthrow—members of one matriline supplant the alpha matriline, typically leading to injury and sometimes death. Matrilineal overthrows can happen at any time of the year, but in 2012, one occurred in a field corral in December, a time in which some females were pregnant with fetuses in their first trimester (n = 13), and others in their second trimester (n = 7) [62]. Once the overthrow was discovered, animals were removed from the corral and were relocated to individual indoor housing (where the infants would become IMR animals). Data from our target sample were compared to data from two control groups, undisturbed animals from a different field corral, and IMR animals from previous years. Animals that experienced the overthrow/relocation events during their first trimester had significantly elevated levels of Emotionality from the responsiveness observations compared to controls, and those that experienced the events during the second trimester displayed more anxious behavior in the human intruder social challenge test. More significant, perhaps, was the finding that the animals exposed during their second trimester had elevated cortisol concentrations, and evidence of a dysregulated HPA axis—there was little differentiation among these animals across the four blood samples. Of course, it is not clear from these data whether the biobehavioral changes were due to the overthrow event itself; the presumed increase in antagonistic interactions leading up to the overthrow; the relocation to individual housing; or to some combination of these experiences. Nevertheless, the data suggest that such events have a lasting impact on biobehavioral organization, at least during the infancy period.

## 4. Discussion

The goal of this review was to describe the CNPRC’s BioBehavioral Assessment Program, and how data from that program have been used in scientific studies. As the precision medicine approach gains influence in medicine, it will become important for animal models to follow suit, and one way of accomplishing this will be to adopt screening programs at facilities where target populations (i.e., those that might be most susceptible to a particular pathogen or drug) can be identified. The BBA program was one of the first screening programs at primate facilities, and the only one of which we are aware where the focus is on biobehavioral characteristics.

We have described two ways in which BBA data have been used. The first is by identifying subgroups of individuals that possess characteristics that one hypothesizes are relevant for the particular process under study. This is a classic personalized medicine approach. Typically, this approach begins with combining BBA data with other existing data related to the process of interest—that is, the first step is often an archival study to show proof of concept. This was the approach taken in our initial study of airway hyperresponsiveness [36], maternal body condition and weight gain during pregnancy [60], social factors relating to autism spectrum disorder, and behavioral inhibition [14]. As it is evident, sometimes, these archival studies lead to publications, but more importantly, they can serve as preliminary data for grant proposals aimed at investigating underlying mechanisms.

A second way that BBA data can be used is in assigning animals to one’s study. As it has been mentioned, experimentalists like to have things controlled. Our investigations into the causes and correlates of variation in biobehavioral measures, described above, provide information on additional factors (e.g., ketamine exposures) that might be worth controlling for in one’s study. BBA data can also be used to ensure experimental and control groups are constructed comparably. To facilitate the use of BBA data for these purposes, at the conclusion of each year’s BBA testing, and after all data have been summarized and checked, they are uploaded to our colony database and are available (along with many hot links describing how the data were generated and why they are important) to everyone who has access to the colony intranet. We facilitate the use of these data by converting most of the measures to z-scores. One might not have any idea what a score of 13.65 for Nervous temperament means, but it is much easier to understand if that value corresponds to a z-score of 3.21—on this measure, the animal’s score is higher than 99.93% of all animals assessed in that year. We have demonstrated that Nervous temperament is associated with glucocorticoid insensitivity [63]. Given that glucocorticoids regulate immune function, if an investigator is planning an infectious disease study, do they really want this animal as a subject? An investigator might want all animals in their study to fall within −1.5 to +1.5 z-units on this measure, for example, and might reject animals outside these boundaries. Once assigned, though, BBA data can then be used to ensure that the study’s groups are reasonably homogeneous, for either behavioral, physiological, or genetic measures. The idea here, then, is that data from programs such as BBA can provide additional information on one’s subjects that can be used to ensure that the groups in one’s study are comparable. Just as one would generally not have all members of the experimental group housed in one room and all members of the control group housed in a separate room, one may also not want the mean score for Nervous temperament in the experimental group to be significantly higher (or lower) than the mean score for the control animals in an infectious disease study.

(We note two additional uses of the BBA program that we have not addressed in this review. The first is that BBA data have been used in colony management. We have published multiple papers on how BBA measures are related to the frequency of stereotypic behavior, diarrhea, and depressive behavior, and to success in social pairings and training using positive reinforcement. These data are reviewed in [8]; see also [64] in this issue. A second additional use of the BBA program is that it provides a highly standardized and comprehensive set of well-characterized biobehavioral assessments that investigators can use in their own studies—that is, in addition to the data, the assessment battery itself has become a resource that numerous investigators have utilized (e.g., [65]).)

To date, BBA data have largely been used by students and colleagues who are aware of, and interested in, the role that psychological factors can play in health and disease. An ongoing issue with the program has been convincing those who are far removed from biobehavioral research that psychological factors are likely important to them as well. For example, there has been little utilization, to our knowledge, of BBA data by infectious disease researchers at our facility, despite the existence of the field of psychoneuroimmunology [66] that clearly indicates that measures such as the ones we quantify in the BBA program influence antibody responses, cellular immune responses, inflammatory processes, etc. This has led to an active outreach program involving presentations describing the program and how it can be used by all investigators. Such efforts must be ongoing to maintain awareness of the program, and the strategy appears to be paying off, with a virologist recently expressing interest in our assessing his animals in an upcoming study. “Selling” the program continues to be an important aspect of our outreach.

Thus far, the focus has been on the use of data from the BBA Program at the CNPRC. What might be the value of the program for investigators outside the CNPRC? Certainly, the knowledge gained from the program, as reflected in hundreds of scientific presentations and dozens of publications, is not specific to the CNPRC and could be applied to other facilities. Second, other facilities might also be interested in implementing a similar program. It is worth noting, however, that the cost of a full 25 h assessment of biobehavioral organization such as the one we have created is high, approximately USD 700 per animal. However, a full replication of BBA is probably not necessary in most cases. Our approach was open-ended: how are intrinsic characteristics associated with management and scientific outcomes? However, based on our results, others may choose to only implement certain assessments, as was the case for another facility that adopted aspects of our program. Deciding on which assessments to adopt is likely to vary from facility to facility, depending on the types of research being conducted. In this regard, we present two general suggestions for further thought. First, many of our results have focused on how our various measures of affect were associated with welfare and scientific outcomes, not surprising given the role that emotion plays in health and well-being [66]. Thus, using assessments that focus on affective outcomes is likely to be most valuable. Second, the data that we obtain on day 2 of the BBA Program have been disproportionately informative, particularly the adaptation observations. While we do see variation in how animals respond to the initial separation and relocation (i.e., the responsiveness observations), how well the animals adapt to the handling and testing procedures is a somewhat different process, one that could be very influential in long-term studies, or in how well the animals adjust to the various colony management routines that they experience. Consequently, having assessments that can tap into longer-term (i.e., longer than simply initial responsiveness) adaptive capabilities may be especially valuable.

A third way that the BBA Program could be of value to external investigators involves our specific data, both for data mining, and for identifying animals that might have characteristics relevant to a remote investigator’s research program. This could lead the investigator to conduct studies at our facility, or to purchase animals with the requisite profile to be studied at the investigator’s facility. In order to facilitate broader usage of our existing data by outside investigators (and students), and to make our protocols accessible, we are developing a website in which this information, including the data, will be available. Finally, the CNPRC breeds animals for sale to other institutions, and investigators at those institutions can obtain access to BBA data if the animals that they purchase were assessed in the BBA Program, or they could specifically request animals that went through the program. This has happened on a handful of occasions already, and we would like to see this become a trend: similar to how age/sex class is an important consideration when purchasing animals, we hope that in the future, in the world of precision medicine, the biobehavioral status of the animals might also be considered valuable, regardless of whether that status is determined from the CNPRC’s BBA Program or from similar screening programs at other institutions.

## 5. Conclusions

Variation in nature is everywhere, and some things that vary can be problematic for experimental behavioral and biological research. The BioBehavioral Assessment Program at the CNPRC, since its inception in 2001, has aimed to quantify variation in biobehavioral factors that could be useful to both colony management staff and scientific staff. Significant progress has been made in the broad areas of biological psychology and psychiatry, with colleagues in our Respiratory Diseases group also being among the more frequent users of the program. The ability to identify subgroups of individuals that are at risk for a poor health outcome, such as asthma, is a hallmark of the growing “personalized medicine” approach to medicine. Moreover, the ability to ensure that one’s experimental groups are balanced on relevant biobehavioral measures contributes to better science by eliminating potential anomalies that sometimes occur with “random” assignment to groups. Finally, programs such as BBA also contribute to the second of the 3Rs, namely, a reduction in animal numbers, by making available already characterized animals from which to choose subjects. This eliminates the need for individual investigators to screen multiple animals to identify subjects of interest, saving time, money, and animal distress.

## Figures and Tables

**Table 1:** Timeline for BBA Program testing.

Time	Activity	Process Being Assessed
Day 1: 0900	Infants delivered	
0915	Holding cage observations	Responsiveness
0945	Novel object placed in cage	Response to novelty
1100	Blood sample #1	HPA regulation
1130	Visual paired comparisons	Memory
1230	Video playback	Response to social challenge
1400	Human intruder	Response to social challenge
1600	Blood sample #2 + dex	HPA regulation
1630	Novel object swapped out	
Day 2: 0700	Holding cage observations	Adaptation
0830	Blood sample #3 + ACTH	HPA regulation
0900	Blood sample #4	HPA regulation
0930	Temperament rating	Temperament
1000	Infants returned to mother	
1100	Mother + infant returned home	
